# Tibiofemoral kinematics: the effect of footwear and foot orthoses during running

**DOI:** 10.1186/1757-1146-6-S1-O17

**Published:** 2013-05-31

**Authors:** Laura Hutchison, Rolf Scharfbillig, Hayley Uden, Chris Bishop

**Affiliations:** 1 School of Health Sciences, Division of Health Sciences, University of South Australia, Adelaide, SA, 5000, Australia

## Background

The knee is the most common site of running related injuries (42.1% of all injuries). Orthoses are thought to manage knee pain by reducing internal tibial rotation as the subtalar joint pronates. However, the influence of the footwear that orthoses are placed in is often ignored. This study aimed to determine the immediate effects of footwear and foot orthoses on transverse plane rotation of the tibiofemoral joint during the stance phase of running.

## Methods

An experimental, within subjects, repeated measures design was used. Three-dimensional tibiofemoral kinematics were estimated in the transverse plane by surface-mounted markers as asymptomatic participants (n = 14) ran in four randomised conditions; neutral shoe, neutral shoe with customised orthoses, neutral shoe with prefabricated orthoses, and a stability shoe. Peak internal/external rotation joint angles and ranges of motion (ROM) during loading response, midstance and propulsion were determined. Immediate subjective comfort was also recorded.

## Results

Significant main effects of condition were observed for all outcomes except tibiofemoral ROM during loading response (*P* < 0.05). All significant differences occurred between the stability shoe and another condition, with less tibiofemoral internal rotation in the stability shoe (mean difference ranged between 1.7° - 6.1°) (*P* < 0.05). The neutral shoe with prefabricated orthoses was reported as more uncomfortable than all other conditions.

## Conclusion

The stability shoe reduced peak tibiofemoral internal rotation throughout stance phase of running more than any other condition. Importantly, it was as comfortable as the other conditions. These results identify the ability for footwear alone to induce proximal kinematic effects.

